# Opioid analgesics: Managing the predictable

**DOI:** 10.1016/j.clinme.2025.100330

**Published:** 2025-05-27

**Authors:** A Barnes, G Heppenstall-Harris, A Dickman

**Affiliations:** a Woodlands Hospice, Liverpool University Hospital Foundation Trust (LUHFT), Liverpool, UK; b Liverpool University Hospital Foundation Trust (LUHFT), Liverpool, UK; c Specialist Palliative Care Services, Liverpool University Hospital Foundation Trust (LUHFT), Liverpool, UK

**Keywords:** Opioids, Analgesia, Morphine, Nausea, Constipation, Sedation, Endocrinopathy, Pruritis, Dependence

## Abstract

It is imperative for clinicians to understand the common adverse effects of opioid therapy and ensure patients are properly counselled on them prior to opioid initiation. Predictable adverse effects are wide ranging, and include bowel dysfunction, nausea, sedation, endocrinopathy and pruritis. Many adverse effects (with the notable exception of constipation) improve with time; however, patients should undergo regular re-assessment to identify new or evolving symptoms. Opioid rotation can also be a useful therapeutic tool to mitigate intolerable opioid adverse effects. This article aims to provide a succinct review of important adverse effects and effective management techniques to enhance patient outcomes and quality of life.

## Highlights

• Opioid-induced constipation is the most common adverse effect of opioid therapy.
• Unlike other symptoms, a tolerance is not built to opioid-induced constipation, therefore symptoms will persist for the duration of opioid treatment.
• Opioids cause nausea by stimulating the chemoreceptor trigger zone (which signals the vomiting centre to undergo emesis), stimulating the vestibular apparatus and also by inducing gastric stasis.
• Opioid-induced sedation can be mitigated by careful titration and appropriate opioid choice and should be monitored for at initiation and dose change.
• Opioid treatment for longer than 1 month can disrupt the hypothalamic–pituitary–adrenal (HPA) and hypothalamic–pituitary–gonadal (HPG) axes, possibly resulting in hypogonadotropic hypogonadism (leading to sexual dysfunction), hypoadrenalism, hyperprolactinaemia and reduced bone mineral density.

## Graphical abstract

**Figure:**
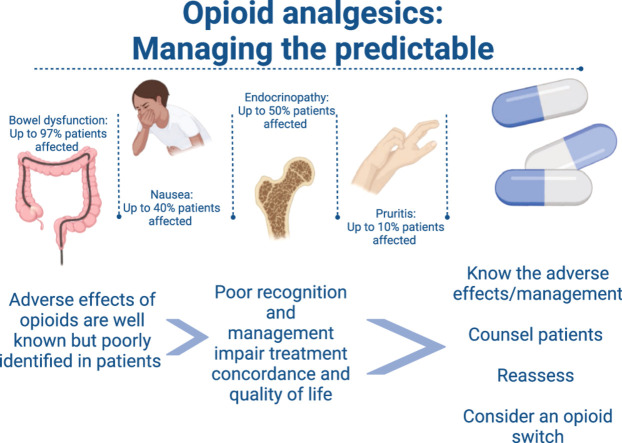
Opioid analgesics: Managing the predictable

## Introduction

Opioids have been used as analgesics for thousands of years, with the earliest recorded use for medicinal purposes dating back to 3400 BC. Opioids remain an invaluable treatment option for cancer-related pain. Despite this, they are known to cause a wide range of predictable adverse effects, such as bowel dysfunction, nausea, sedation, endocrinopathy and pruritis. Patients should be informed of these effects prior to opioid initiation, and monitored for them thereafter.^1^

## Opioid-induced constipation

Opioid-induced constipation (OIC) is defined as new or escalating symptoms of constipation when starting or amending opioid therapy.^2^ It is the most common form of opioid-induced bowel dysfunction, affecting up to 97% of people prescribed opioids for cancer-related pain.^3^

Opioids induce constipation by binding to μ-receptors in the gastrointestinal tract. This has an inhibitory effect on the neurotransmitters serotonin, acetylcholine and substance P, causing increased water reabsorption from the bowel, and reduced smooth muscle contraction.^1^ This neurotransmitter inhibition results in sluggish bowels; hard, dry stools, and increased anal sphincter and ileocaecal valve tone. This all contributes to constipation.

The clinical features of OIC include reduced frequency of spontaneous evacuation, tenesmus and straining. Despite OIC being a universally well-known adverse effect of opioid therapy, it is still often poorly recognised and managed in the clinical setting.^3^ Diagnosis should involve a thorough history and examination, with reference to the Rome IV criteria for OIC (Table 1) to maximise identification of more subtle symptoms.

**Table 1:** Rome IV criteria for opioid-induced constipation.^2^^,^^3^

1. New, or worsening, symptoms of constipation when initiating, changing or increasing opioid therapy, which must include two or more of the following:
Straining during more than 25% of defecations
Lumpy or hard stools (Bristol 1–2) more than 25% of defecations
Sensation of incomplete evacuation for more than 25% of defecations
Sensation of anorectal obstruction/blockage more than 25% of defecations
Manual manoeuvres to facilitate bowel evacuation for more than 25% of defecations
Fewer than three spontaneous bowel motions per week
2. Also must include:
Loose stools are rarely present without the use of laxatives

The National Institute for Health and Care Excellence (NICE) recommends that a stimulant laxative should be prescribed upon commencement of an opioid.^1^ The addition of an osmotic laxative is recommended if there has been a lack of response within 3–4 days to a stimulant laxative that has been titrated to maximum dose. Rectal interventions such as enemas and suppositories are only recommended if the above has been ineffective, and there is clinical evidence of stool in the rectum or descending colon.^3^^,^^4^

Peripherally acting μ-receptor antagonists (PAMORAs) such as naloxegol and naldemedine are a useful treatment for OIC that has failed to respond to conventional laxative therapy. The Multinational Association for Supportive Care in Cancer (MASCC) guidelines recommend that PAMORAs are always considered in the management of patients with OIC.^3^

Importantly, unlike other opioid adverse effects, tolerance does not develop to OIC because the colon does not adapt to the presence of opioids in the same way that the central nervous system (CNS) does. OIC therefore persists for the duration of opioid therapy.^4^^,^^5^ This predictable adverse effect can significantly reduce patients’ quality of life and compliance with treatment.^5^ OIC therefore requires prophylactic management, prompt identification and thorough reassessment throughout the duration of opioid therapy.

## Opioid-induced nausea and vomiting

Opioid-induced nausea and vomiting (OINV) is another common adverse effect of opioid therapy, affecting approximately 25–40% of patients.^6^

Opioids trigger nausea by stimulating the chemoreceptor trigger zone (CTZ), located at the base of the fourth ventricle and positioned partly outside the blood–brain barrier. The CTZ sends signals to the medullary vomiting centre, which initiates the vomiting reflex. Opioids also directly stimulate the vestibular apparatus, replicating the sensation of motion sickness. Notably, with continued use, the CNS adapts to the presence of opioids, reducing the effects described above over time.^6^ This is important to highlight to patients, as the initial emetogenic effects often subside spontaneously in the days after initiation. Peripherally, gastrointestinal motility is inhibited by μ-receptor agonism, leading to gastric stasis and delayed gastric emptying, contributing to OINV. A degree of tolerance develops to gastric stasis over time.^7^

NICE recommends that, if nausea persists, anti-emetic therapy should be prescribed and optimised prior to considering an opioid switch. Changing the opioid can be a useful management strategy after anti-emetic optimisation has failed.^1^

OINV is often multifactorial and therefore management should be individualised by thorough patient assessment of probable cause, non-opioid-related factors and patient comorbidity (Table 2).

**Table 2:** An approach to appropriate anti-emetic selection in OINV based on pathophysiology.^6^^,^^8^

Pathophysiology of nausea and related neurotransmitter	Related symptoms/signs	Anti-emetic and mechanism of action
Gastric stasis Dopamine via peripheral D_2_ gastric receptors	Early satiety, bloating, postprandial worsening, oesophageal reflux, infrequent vomiting, hiccups	Metoclopramide (NICE recommended first line for gastric stasis) D_2_ receptor antagonist at CTZ and peripheral D_2_ receptors Domperidone D_2_ receptor antagonist at CTZ and peripheral D_2_ receptors Haloperidol D_2_ receptor antagonist at CTZ and peripheral D_2_ receptors
Chemoreceptor trigger zone stimulation Dopamine via D_2_, serotonin via 5HT_3_ Neurokinin via NK-1 receptor	Chronological relation to new drug/toxin, constant nausea	Haloperidol D_2_ receptor antagonist at CTZ Domperidone D_2_ receptor antagonist at CTZ Ondansetron/granisetron 5HT3 receptor antagonist Aprepitant Neurokinin-1 receptor antagonist
Vestibular apparatus stimulation Histamine via H_1_ receptor, Acetylcholine via anti-muscarinic receptor	Motion-associated emesis, vertigo	Cyclizine H1 receptor antagonist Hyoscine hydrobromide Central muscarinic receptor antagonist

OINV is common, and often incorrectly identified as a sensitivity reaction, rather than an expected pharmacological response which should settle in the days following opioid initiation. OINV can be a highly distressing symptom, so adequate patient counselling and individualised anti-emetic selection can improve compliance, enteral absorption and quality of life.^8^

## Opioid-induced sedation

Opioid-induced sedation (OIS) can affect around 40% of people taking an opioid for cancer pain and most commonly occurs as an acute adverse effect at initiation or dose titration.^9^ Sedation occurs because opioids cause depression of the reticular activating system in the brainstem, dysregulating the sleep/wake cycle. Patients develop a tolerance to the sedative effects of opioids over time.^10^

Patients should be counselled about the risk of sedation, especially with regards to driving or operating heavy machinery. Careful opioid selection can mitigate comorbid factors that may worsen sedation, such as renal impairment and co-prescription with other sedating medications. Should the sedation persist, clinicians should consider an opioid switch. There is weak evidence for the use of psychostimulants like methylphenidate.^11^

OIS may present in a patient on stable opioid therapy if there are changes to opioid pharmacokinetics, the pain resolves (for example, following addition of an adjuvant analgesic, or an interventional pain procedure) or the opioid is taken in overdose. Opioid toxicity can present with acute sedation, in addition to confusion, respiratory depression and myoclonus. Toxicity is feared by patients and clinicians alike; however, while causing serious adverse effects, it should not occur if opioid titration is undertaken carefully, and regularly reviewed.^10^

## Opioid-induced endocrinopathy

Endocrinopathies associated with opioids are commonly encountered, but often under-reported. The prevalence of hypogonadism is variable, but estimated to be up to 50% in people with chronic opioid use.^12^ The severity of all associated endocrinopathies is dependent on the type, strength, form and route of opioid medication.

Long-term opioid use disrupts the hypothalamic–pituitary–adrenal (HPA) and hypothalamic–pituitary–gonadal (HPG) axes. Opioids directly inhibit the pulsatile release of gonadotropin-releasing hormone (GnRH) from the hypothalamus, which reduces the secretion of luteinising hormone (LH) and (to a lesser extent) follicle-stimulating hormone (FSH) from the pituitary gland. This leads to reduced gonadal sex steroid production, causing hypogonadism and also negatively impacting bone metabolism, increasing osteoporosis risk.^13^ Hypogonadism often presents with sexual dysfunction; however, this is often under-recognised as an opioid adverse effect, due in part to barriers discussing sexual function.^14^ The inhibitory endocrine effects begin upon opioid commencement, and drug cessation results in axis recovery.

Chronic opioid use is also an independent risk factor for secondary adrenal insufficiency, the prevalence of which is less well defined. Opioids suppress the release of corticotropin-releasing hormone (CRH) from the hypothalamus and adrenocorticotropic hormone (ACTH) from the pituitary, therefore reducing cortisol production in the adrenal gland.

There is a spectrum of presentations related to endocrinopathy, notably sexual dysfunction, fatigue, low mood and fragility fractures. A thorough history and examination should be used to identify signs of hypogonadism, hypoadrenalism, hyperprolactinaemia and low bone mineral density, and rule out non-opioid-related causes (Table 3).

**Table 3:** Approach to monitoring and management of opioid-induced endocrinopathies.^12^, ^13^, ^14^, ^15^, ^16^

Endocrinopathy	Investigation	Management strategy
Hypogonadotropic hypogonadism	Men: Serum testosterone, FSH, LH, sex hormone-binding globulin (SHBG) Women: Serum oestradiol, FSH, LH	Consider opioid discontinuation or switch or gonadal hormone replacement therapy
Hypoadrenalism	Early morning serum cortisol	Consider opioid reduction or discontinuation AND glucocorticoid replacement therapy
Hyperprolactinaemia	Serum prolactin	Consider opioid switch or discontinuation
Reduced bone mineral density	Dual-energy X-ray absorptiometry (DXA) scan	Consider opioid discontinuation AND management of osteopenia/ osteoporosis

## Opioid-induced pruritis

Pruritis is a less common but troublesome adverse effect of opioids; however, the underlying cause is less well understood. Opioid medication acts both centrally and peripherally to cause itching through various excitatory neurotransmitters, including histamine release. Morphine and codeine cause the most significant histamine release.^17^ Measures such as menthol-based creams and H_1_ receptor antagonists have a role in the management of itch, as do 5HT3 antagonists, D2 receptor antagonists and GABA antagonists.

## Opioid dependence

Clinicians often have concerns about addiction risk when prescribing opioids. In practice, addiction and dependence are different concepts. Dependence is an expected physiological response to adaptations seen with regular opioid use, whereas addiction is a complex biopsychosocial syndrome involving compulsive substance use despite harmful consequences. In advanced illness, the priority should be ensuring adequate pain control. Patients may develop a physiological tolerance to opioids; however, addiction is rarely an issue.^10^ Patients with premorbid opioid misuse may require different management and should receive specialist palliative medicine input.^18^

## Opioid switching

Individualised prescribing should be used to select the most appropriate opioid, with factors such as comorbidity (eg renal or liver impairment) and concomitant medication being considered. Partial μ-receptor agonists like buprenorphine have been shown to cause fewer adverse effects compared with full μ-receptor agonists like morphine (traditionally, the first-line opioid for cancer pain).^19^ Selecting the most appropriate opioid to switch to should be undertaken after comprehensive patient assessment and advice from palliative medicine clinicians.

## Conclusion

It is imperative for clinicians to understand the common and debilitating adverse effects of opioid use, and to ensure that patients are properly counselled prior to opioid initiation, and regularly reassessed. Many adverse effects (with the notable exception of constipation) improve with time. All patients should be prescribed a laxative on commencement of opioid therapy.^1^ If nausea remains an issue after opioid commencement, an assessment of likely cause is recommended to ensure that targeted anti-emetic therapy is prescribed.

Opioid switch can be a useful therapeutic tool to mitigate intolerable opioid adverse effects. An understanding of opioid pharmacology, in addition to considering individualised patient factors, should be used to identify which opioid would be the most appropriate for individual patients.

## Funding

This research did not receive any specific grant from funding agencies in the public, commercial or not-for-profit sectors.

## CRediT authorship contribution statement

**A Barnes:** Writing – review & editing, Writing – original draft, Visualization, Project administration. **G Heppenstall-Harris:** Writing – review & editing, Writing – original draft. **A Dickman:** Writing – review & editing, Writing – original draft, Visualization, Supervision, Project administration.

## Declaration of competing interests

The authors declare the following financial interests/personal relationships which may be considered as potential competing interests: Andrew Dickman reports a relationship with Sandoz Inc that includes: consulting or advisory and speaking and lecture fees. Andrew Dickman reports a relationship with Kyowa Kirin Inc that includes: consulting or advisory and speaking and lecture fees. If there are other authors, they declare that they have no known competing financial interests or personal relationships that could have appeared to influence the work reported in this paper.

## References

[1] Overview | palliative care for adults: strong opioids for pain relief | guidance. NICE 2016 [cited 26 March 2025]. Available from: https://www.nice.org.uk/guidance/cg140.

[2] Mearin F., Lacy B.E., Chang L. (2016). Bowel disorders. Gastroenterology.

[3] Davies A., Leach C., Caponero R. (2020). MASCC recommendations on the management of constipation in patients with advanced cancer. Support Care Cancer.

[4] Prescribing in palliative care. [cited 26 March 2025]. NICE. Available from: https://bnf.nice.org.uk/medicines-guidance/prescribing-in-palliative-care/

[5] Akbarali H.I., Inkisar A., Dewey W.L (2014). Site and mechanism of morphine tolerance in the gastrointestinal tract. Neurogastroenterol Motil.

[6] Smith H.S., Smith J.M., Seidner P. (2012). Opioid-induced nausea and vomiting. Ann Palliat Med.

[7] Kurz A., Sessler D.I (2003). Opioid-induced bowel dysfunction: pathophysiology and potential new therapies. Drugs.

[8] Palliative care - nausea and vomiting: prescribing a prokinetic. [cited 26 March 2025]. NICE. Available from: https://cks.nice.org.uk/topics/palliative-care-nausea-vomiting/prescribing-information/prescribing-a-prokinetic/

[9] Lim K.-H., Nguyen N.-N., Qian Y. (2018). Frequency, outcomes, and associated factors for opioid-induced neurotoxicity in patients with advanced cancer receiving opioids in inpatient palliative care. J Palliat Med.

[10] Vella-Brincat J., Macleod A.D (2007). Adverse effects of opioids on the central nervous systems of palliative care patients. J Pain Palliat Care Pharmacother.

[11] McNicol E., Horowicz-Mehler N., Fisk R.A. (2003). Management of opioid side effects in cancer-related and chronic noncancer pain: a systematic review. J Pain.

[12] Fountas A., Van Uum S., Karavitaki N. (2020). Opioid-induced endocrinopathies. Lancet Diabetes Endocrinol.

[13] Seyfried O., Hester J. (2012). Opioids and endocrine dysfunction. Br J Pain.

[14] Salata B., Kluczna A., Dzierżanowski T. (2022). Opioid-induced sexual dysfunction in cancer patients. Cancers (Basel).

[15] Adrenal insufficiency. [cited 26 March 2025]. NICE Available from: https://bnf.nice.org.uk/treatment-summaries/adrenal-insufficiency/

[16] Osteoporosis - prevention of fragility fractures: scenario: management. NICE. [cited 26 March 2025]. Available from: https://cks.nice.org.uk/topics/osteoporosis-prevention-of-fragility-fractures/management/management/

[17] Okutani H., Lo Vecchio S., Arendt-Nielsen L. (2024). Mechanisms and treatment of opioid-induced pruritus: peripheral and central pathways. Eur J Pain.

[18] Singh S.A., Moreland R.A., Fang W., Shaikh P., Perez J.M., Morris A.M. (2021). Compassion inequities and opioid use disorder: a matched case-control analysis examining inpatient management of cancer-related pain for patients with opioid use disorder. J Pain Symptom Manage.

[19] Gudin J., Fudin J. (2020). A narrative pharmacological review of buprenorphine: a unique opioid for the treatment of chronic pain. Pain Ther.

